# Supplementary material for: Ultrabright and stable top-emitting quantum-dot light-emitting diodes with negligible angular color shift
Source: Nat Commun. 2024 Jun 17;15:5161. doi: 10.1038/s41467-024-49574-6 (PMC11183122; doi:10.1038/s41467-024-49574-6)
Supplement: Supplementary file 1 — Supplementary Information [file 41467_2024_49574_MOESM1_ESM.pdf]

Supplementary Information

Ultrabright and Stable Top-Emitting Quantum-Dot Light-Emitting Diodes with Negligible Angular Color Shift

Mengqi Li,[1] Rui Li,[1] Longjia Wu,[2] Xiongfeng Lin,[2] Xueqing Xia,[1] Zitong Ao,[2] Xiaojuan Sun,[1] Xingtong Chen,[1] and Song Chen[1,3,*]

[1] Suzhou Key Laboratory of Novel Semiconductor-optoelectronics Materials and Devices, College of Chemistry, Chemical Engineering and Materials Science, Soochow University, Suzhou 215123, Jiangsu, China.

[2] TCL Corporate Research, 1001 Zhongshan Park Road, Nanshan District, Shenzhen 518067, Guangdong, China

[3]Jiangsu Key Laboratory of Advanced Negative Carbon Technologies, Soochow University, Suzhou, 215123, Jiangsu, P.R. China

*Correspondence: songchen@suda.edu.cn

Supplementary Note 1. Calculation of optical penetration depths.

To achieve resonance conditions in single-mode devices, the total optical length of the microcavity should be half the wavelength of the QD emission：

$$L_{\mathrm{cav}} = \frac{\lambda}{2} = 268.5\ \mathrm{nm} \tag{1}$$

Previous literature generally used the following expression to calculate phase shifts[1,2]:

$$\varphi = \arctan\frac{2n_0k_1}{n_0^2 - n_1^2 - k_1^2} \tag{2}$$

where n_0 is the refractive index of the dielectric medium, n_1 and k_1 represent the real and imaginary parts of the metal's refractive index, respectively. According to Equation (2) and Supplementary Table. 1, the phase shifts at the top and bottom mirrors are：

$$\varphi_1 = \arctan\frac{2n_0k_1}{n_0^2 - n_1^2 - k_1^2} = \arctan\frac{2\times1.55\times3.07}{1.55^2 - 0.133^2 - 3.07^2} = -0.93 \tag{3}$$

$$\varphi_2 = \arctan\frac{2n_0k_1}{n_0^2 - n_1^2 - k_1^2} = \arctan\frac{2\times1.93\times3.32}{1.93^2 - 0.05^2 - 3.32^2} = -1.05 \tag{4}$$

The corresponding penetration depths at the top and bottom of the optical cavity are calculated as：

$$L_{\mathrm{pen1}} = \frac{\lambda(\pi - \varphi_1)}{4\pi} = \frac{537\times[3.1416 - (-0.93)]}{4\times3.1416}\ \mathrm{nm} = 174.0\ \mathrm{nm} \tag{5}$$

$$L_{\mathrm{pen2}} = \frac{\lambda(\pi - \varphi_2)}{4\pi} = \frac{537\times[3.1416 - (-1.05)]}{4\times3.1416}\ \mathrm{nm} = 179.1\ \mathrm{nm} \tag{6}$$

Then, the total electrode penetration depth is about 350 nm, which is clearly not compatible with the total cavity length obtained in Equation (1).

Considering the optical constant characteristics of metals, the phase shift should be located in the second quadrant. An extra phase shift of π should be added to Equation (2), Equation (3) and (4) should be corrected to：

$$\varphi_1 = \pi + \arctan\frac{2n_0k_1}{n_0^2 - n_1^2 - k_1^2} = \pi + \arctan\frac{2\times1.55\times3.07}{1.55^2 - 0.133^2 - 3.07^2} = 2.21 \tag{7}$$

$$\varphi_2 = \pi + \arctan\frac{2n_0k_1}{n_0^2 - n_1^2 - k_1^2} = \pi + \arctan\frac{2\times1.93\times3.32}{1.93^2 - 0.05^2 - 3.32^2} = 2.09 \tag{8}$$

According to Equation (7) and (8), the penetration depths into the top and bottom mirrors are：

$$L_{\text{pen1}} = \frac{\lambda(\pi - \varphi_1)}{4\pi} = \frac{537\times(3.1416 - 2.21)}{4\times3.1416}\ \text{nm} = 39.8\ \text{nm} \tag{9}$$

$$L_{\text{pen2}} = \frac{\lambda(\pi - \varphi_2)}{4\pi} = \frac{537\times(3.1416 - 2.09)}{4\times3.1416}\ \text{nm} = 44.9\ \text{nm} \tag{10}$$

The total penetration depth (~85 nm) is compatible with the single-mode cavity.

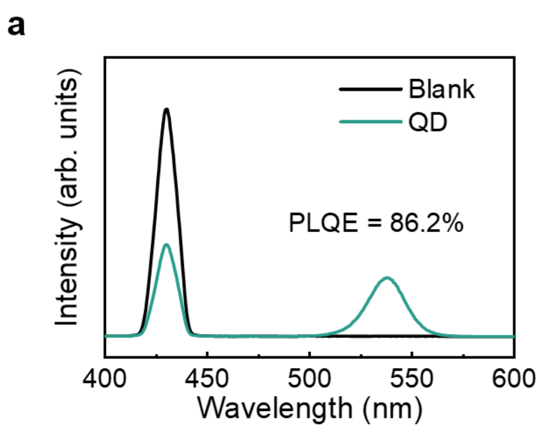

Supplementary Fig. 1 | Photoluminescence quantum efficiency, PLQE. a The PLQE measured from QD solution (solvent: Octane). **b** The external PLQE, η_{ext}, measured from QD thin films and the fitted internal PLQE, η_{int}. The three thin film samples were deposited on different metal substrates (Ag, Al, Au) to enable the fitting of η_{int}. η_{int} was calculated by equation[3]: $\eta_{ext} = \frac{\eta_{int}/2n^2}{\eta_{int}/2n^2+(1-\eta_{int})+L/4\alpha_0 d_0}$, where n and α_0 are the average refractive index and the average band-edge absorption coefficient (1.68×10^4 cm^{-1}), d_0 is the film thickness (40 nm). L is the parasitic loss of the back-reflecting mirror (L = $1-R$, R is the reflectivity of the metal substrate). Such a method evaluates the internal PLQE of solid films by considering the loss due to reflection, absorption, and waveguide. For comparison, the QD film deposited on a bare glass substrate exhibits a PLQE of 67%, indicating the existence of optical loss.

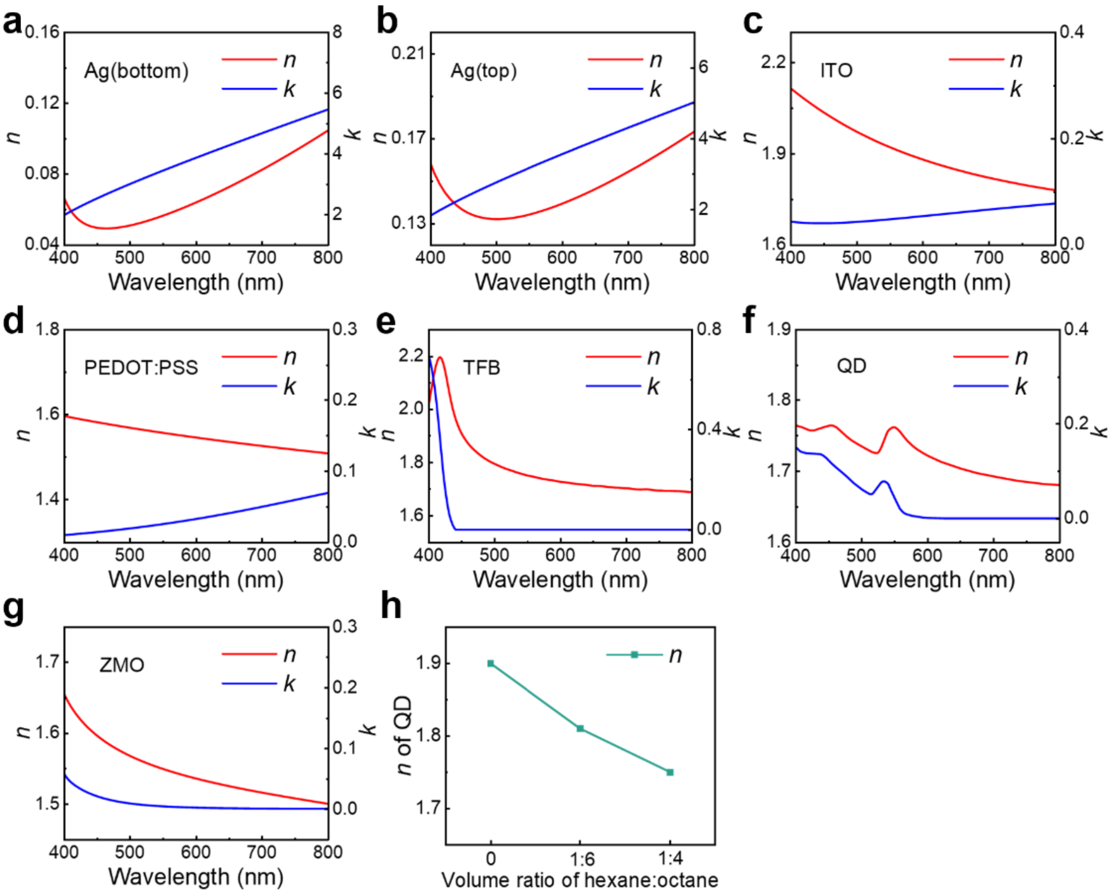

Supplementary Fig. 2 | Refractive indices (*n*, i*k*) of the thin film materials used to fabricate a TE QLED. a Silver (bottom mirror). **b** Silver (top mirror). **c** ITO (optical spacer). **d** PEDOT:PSS (hole-injection layer). **e** TFB (hole-transporting layer). **f** Green-emitting colloidal quantum dots (emission layer). **g** ZnMgO (electron-transporting layer). **h** The effect of mixed solvent on the refractive indices of QD films, The films subjected to low vacuum (~1×10^{-3} Pa) processing for 30 minutes.

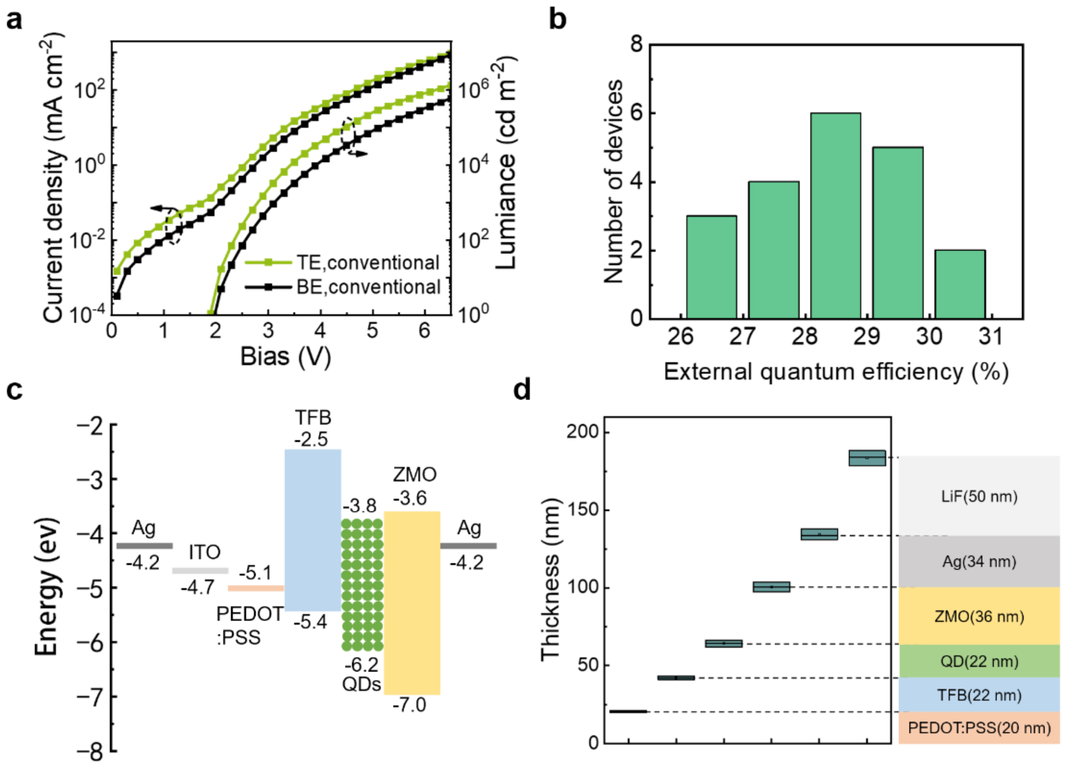

Supplementary Fig. 3 | Device performance and structure. a TE and BE devices fabricated with the conventional process. Because the QD and ZMO layers are much thinner than necessary, the TE devices generally show high leakage current, low efficiency, and high failure rate during regular tests. **b** EQE distribution of TE devices with optimal parameters (measured from 20 devices). **c** Schematic diagram of energy level arrangement of TE devices. **d** Layer stack thickness measured by AFM after depositing each functional layer. The box widths denote the range of three data points. The ITO/Ag/ITO stacks below the PEDOT:PSS layer were fabricated and thickness calibrated in a TCL CSOT fab.

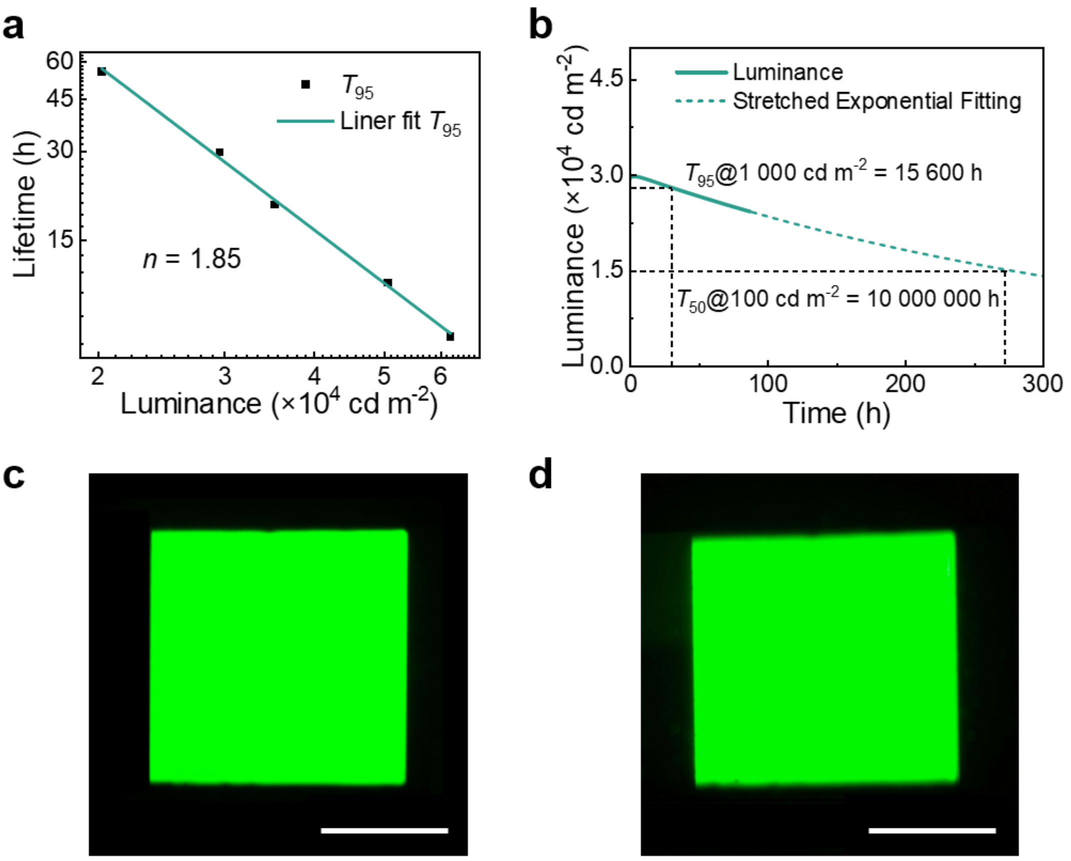

Supplementary Fig. 4 | Operational lifetime. a Fitting of accelerating factor (n) using the equation: $L_0^n \cdot T_{95}$ = constant. **b** Operational lifetime of TE QLEDs. The current density at 100 and 1000 cd m^{-2} are 0.2 and 1.1 mA cm^{-2}, respectively. The extrapolated curve and the T_{50} value were obtained by fitting the measured decay curve using stretched exponential decay functions[4,5]. **c** The image of the emissive area before the lifetime test (scale bar = 1 mm). **d** The image of the emissive area after the lifetime test (scale bar = 1 mm).

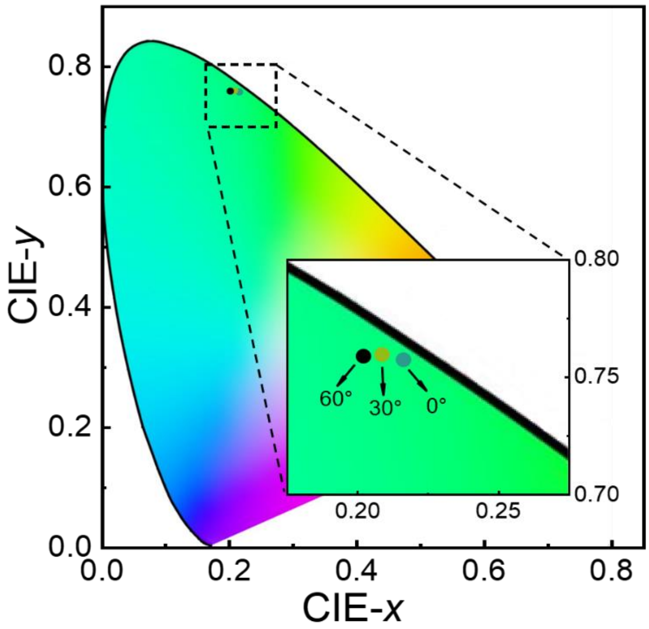

Supplementary Fig. 5 | Angular color shift. Color coordinates in the CIE 1931 non-uniform diagram (*x*,*y*) of the TE QLED (QD FWHM = 21 nm) from 0° to 60°. In CIE-1931 (*x*,*y*), the green colors appear to have larger shifts than the red and blue colors because of the uneven distribution of colors. As a result, this diagram is less recommended for studying color shifts.

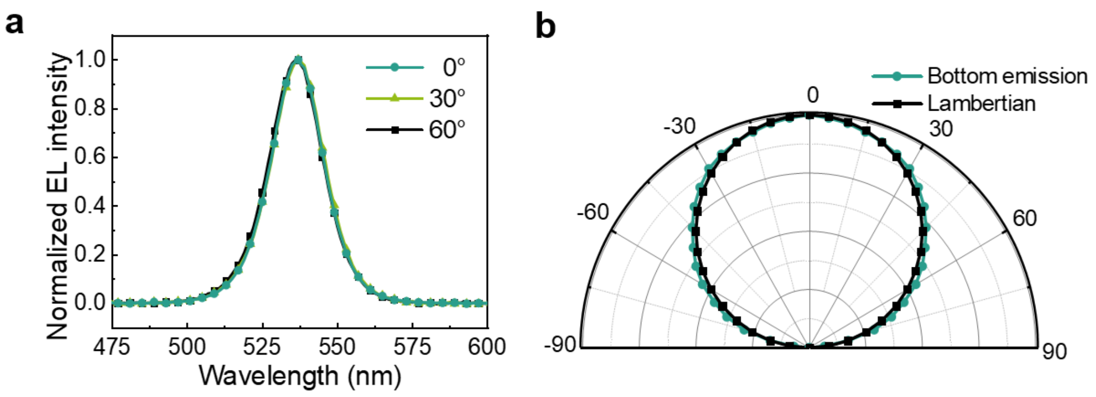

Supplementary Fig. 6 | Angular dependence of BE QLEDs. a EL spectra measured from the off-axis angle of 0° to 60°. **b** Normalized emission intensity measured at different off-axis angles.

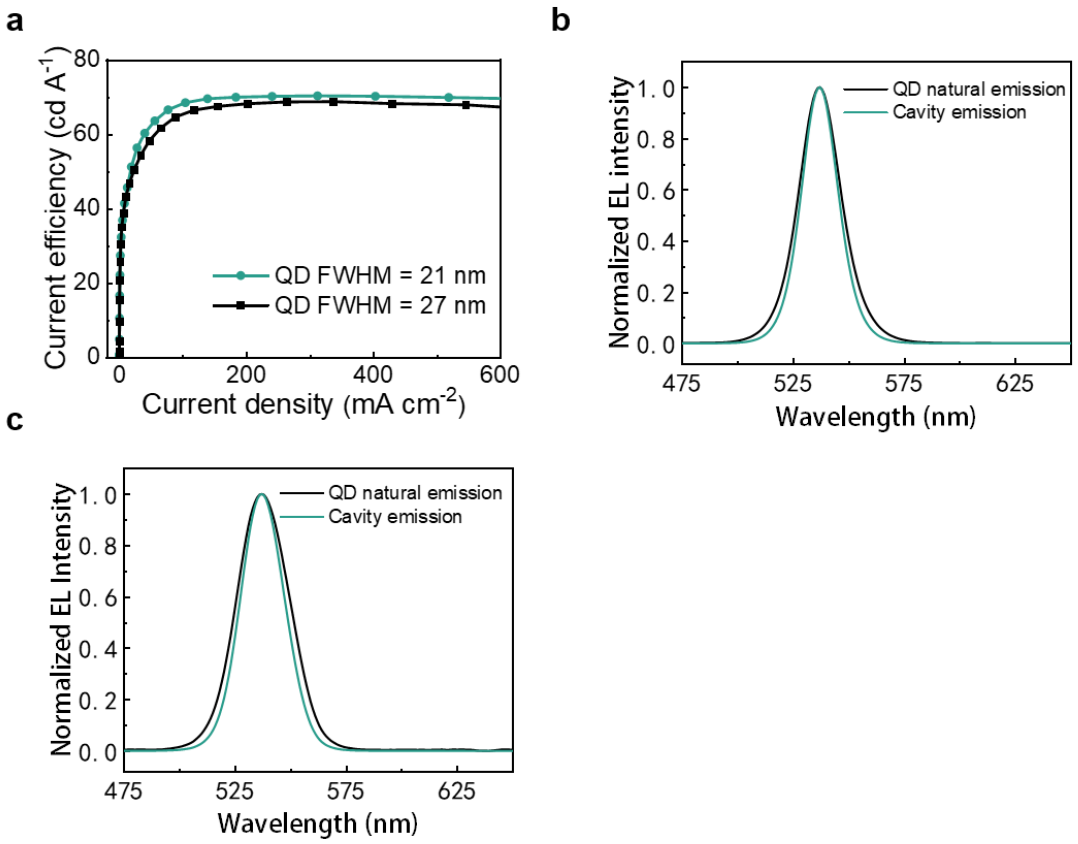

Supplementary Fig. 7 | Emission linewidths. a Current efficiency of BE QLEDs with different emission linewidths. **b** Simulated microcavity emission spectrum (FWHM = 19.4 nm) and QDs' natural emission spectrum (FWHM = 21 nm). **c** Simulated microcavity emission spectrum (FWHM = 22.8 nm) and QDs' natural emission spectrum (FWHM = 27 nm). For comparison, QDs with an FWHM of 21 nm (or 27 nm) offer an FWHM of 19.2 nm (or 22.3 nm) in real devices. The angular color shifts can be estimated using the simulated optical mode and QDs' natural emission spectra. The results show an angular color shift of $\Delta u'v'$ = 0.0086 (QDs' FWHM = 27 nm) and 0.0060 (QDs' FWHM = 21 nm), which are consistent with the results measured from real devices.

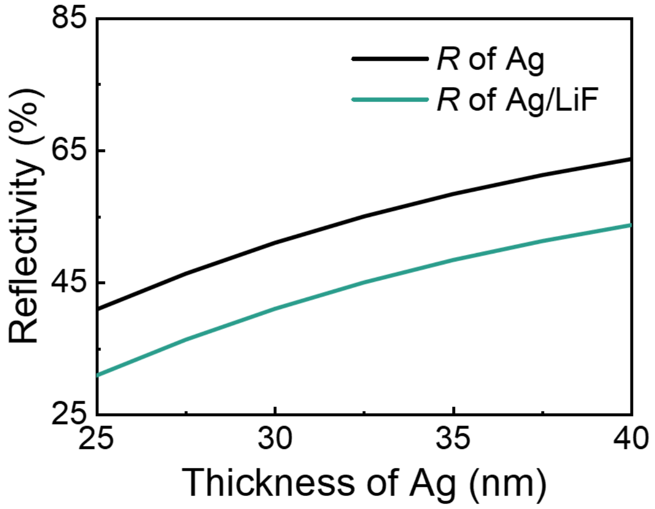

Supplementary Fig. 8 | Reflectivity of Ag and Ag/LiF (50 nm) as a function of the silver layer's thicknesses.

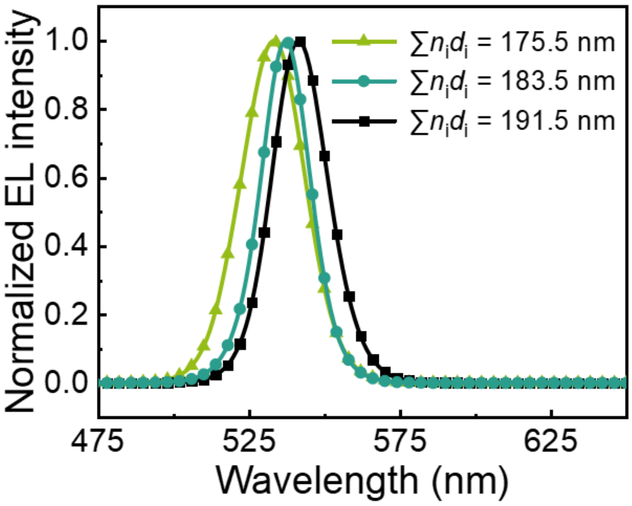

Supplementary Fig. 9 | EL spectra of TE QLEDs with different cavity lengths. The change in cavity length is due to the change in the semiconducting layers' thickness.

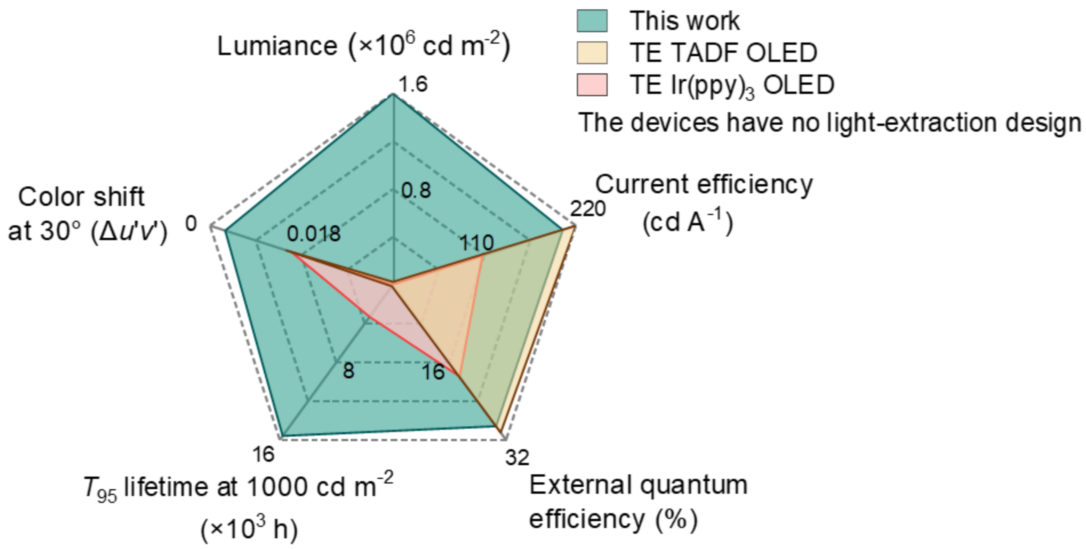

Supplementary Fig. 10 | Comparison of device performance with benchmark TE OLEDs[6,7]. Color shift or lifetime data was not provided in references 6 or 7. For the integrity of the OLED data, we relied on the result reported elsewhere from a similar device (see ref. 8-10). The comparison does not consider devices adopting light extraction methods, including microlens arrays, scattering layers, and corrugated structures.

Supplementary Table. 1 | Complex refractive index and thickness of each functional layer in Fig. 4d-f.

Material	N=n+ik (λ = 537 nm)	Fig. 4d (nm)	Fig. 4e (nm)	Fig. 4f (nm)
Ag (bottom)	0.05+3.32i	110	110	110
Ag (top)	0.133+3.07i	30/34/38	34	34
ITO	1.93	10	10	10
PEDOT:PSS	1.56	20	18/20/22	17/20/22
TFB	1.76	22	22	22
QDs	1.75	22	22	22
ZnMgO	1.55	36	33/36/39	39/36/34

Supplementary Table. 2 | Comparison of the device performance with benchmarking QLEDs.

Device	Year (ref)	Structure	EL Peak (nm)	FWHM (nm)	CIE-1931(x,y) at 0°	$\Delta u'v'$ at 30°/60°	V_{on} (V)	L_{max} (cd m^{-2})	EQE (%)	CE (cd A^{-1})	T_{95}@1,000 cd m^{-2} (h)	T_{95}@100 cd m^{-2} (h)	T_{50}@100 cd m^{-2} (h)
BE (Green)	2018(ref. 11)	ITO/ZnO/PVK/QD/PEIE/poly-TPD/MoOx/Al	525	20	(0.13,0.79)	--/--	5.75	72,814	22.4	89.8	--	--	--
	2019(ref. 12)	ITO/PEDOT:PSS/TFB/QDs/ZnO/Al	534	44.5*	(0.34,0.64)*	0.0018* /0.0035*	2.0	614,000	22.9	98*	4,200*	280,000*	1,760,000
	2020(ref. 13)	ITO/PEDOT:PSS/TFB/QDs/ZnO/Al	530	26	(0.22,0.73)	--/--	2.2	13,200	23.9	100.5	2,500	147,000*	1,655,000
	2021(ref. 14)	ITO/PEDOT:PSS/TFB/QDs/ZnO/Al	525	27	--	--/--	2.1	485,000	22.9	98.4	--	--	--
	2022(ref. 15)	ITO/PEDOT:PSS/PF8Cz/QDs/ZnMgO/Al	537	26	(0.29,0.53)*	--/--	2.05	210,000	28.7	127	7,200	580,000	2,570,000
	2023(ref. 16)	ITO/PEDOT:PSS/TFB/QDs/ZnMgO/Al	527	23	(0.14,0.78)	0.0017* /0.0021*	2.2	100,000	20.1	79.8	--	--	--
TE (Red/ (Orange-red)	2016(ref. 17)	Ag/ZnO/QDs/TCTA/NPB/HCTCN/Ag (multi-mode)	632	26	(0.70,0.30)	0.0350* /0.0613*	3.1	112,000	10.1	27.8	--	--	--
	2021(ref. 18)[a)]	Ag/ZnO/QDs/CBP/MoO_x/HAT-CN/Ag (single-mode)	615	17	(0.64,0.36)*	--	2.5	650,000	14.7	55.6	85,000*	5,360,000*	12,600,000
TE (Green)	2017(ref. 19)	Al/MoO_3/PVK/QDs/ZnO/Ag (multi-mode)	538	30	(0.26,0.71)	0.0082*/--	4.9	151,000	7.4	33.7	1.4*	110*	9,691
	2019(ref. 20)	Ag/ZnO/PFN/QDs/CzSi/TCTA/MoO_X/Ag (multi-mode)	539	37	(0.25,0.72)*	--/--	2.3	17,400	3.4	21.6	--	--	--
	2021(ref. 21)	Ag/Glass/ITO/PVK/TFB/Perovskite/TPBi/LiF/Mg:Ag/MoO_3 (multi-mode)	512	19.9*	(0.09,0.76)*	0.0042* /0.0061*	2.8	12,000	16.1	51.5	--	--	--
	**This Work**	**ITO/Ag/ITO/PEDOT:PSS/TFB/QDs /ZnMgO/Ag/LiF (single-mode)**	**537**	**19.2**	**(0.21,0.76)**	**0.0030 /0.0052**	**1.9**	**1,600,000**	**29.2**	**204.2**	**15,600**	**1,100,000**	**10,000,000[b)]**

-- data not provided; * data extracted from the published plot;

[a)] data for devices on silicon substrates is not included; [b)] data obtained using stretched exponential fitting

Supplementary References

1. Miao, Y. et al. Microcavity top-emission perovskite light-emitting diodes. *Light Sci. Appl.* **9**, 89 (2020).
2. Deng, Z. B. et al. Optical microcavity based on porous and organic materials. *Synth. Met.* **129**, 299-302 (2002).
3. Schnitzer, I., Yablonovitch, E., Caneau, C. & Gmitter, T. J. Ultrahigh spontaneous emission quantum efficiency, 99.7% internally and 72% externally, from AlGaAs/GaAs/AlGaAs double heterostructures. *Appl. Phys. Lett.* **62**, 131-133 (1993).
4. Fery, C., Racine, B., Vaufrey, D., Doyeux, H. & Cina, S. Physical mechanism responsible for the stretched exponential decay behavior of aging organic light-emitting diodes. *Appl. Phys. Lett.* **87**, 213502 (2005).
5. Sebastian, S., Denis, K., Karl, L. & Björn, L. Degradation mechanisms and reactions in organic light-emitting devices. *Chem. Rev.* **115**, 8449-8503 (2015).
6. Zhang, Y. et al. Fusion of multi-resonance fragment with conventional polycyclic aromatic hydrocarbon for nearly BT.2020 green emission. *Angew. Chem. Int. Ed.* **61**, 202202380 (2022).
7. Pyo, B. et al. A nanoporous polymer film as a diffuser as well as a light extraction component for top emitting organic light emitting diodes with a strong microcavity structure. *Nanoscale* **8**, 8575-8582 (2016).
8. Zhang, D., Wei, P., Zhang, D. & Duan, L. Sterically shielded electron transporting material with nearly 100% internal quantum efficiency and long lifetime for thermally activated delayed fluorescent and phosphorescent OLEDs. *ACS Appl. Mater. Interfaces* **9**, 19040-19047 (2017).
9. Fukagawa, H., Oono, T., Iwasaki, Y., Hatakeyama, T. & Shimizu, T. High-efficiency ultrapure green organic light-emitting diodes. *Mater. Chem. Front.* **2**, 704-709 (2018).
10. Sudheendran S, S. et al. Approaches for long lifetime organic light emitting diodes. *Adv. Sci.* **8**, 2002254 (2020).

11. Fu, Y., Jiang, W., Kim, D., Lee, W. & Chae, H. Highly efficient and fully solution-processed inverted light-emitting diodes with charge control interlayer. *ACS Appl. Mater. Interfaces* **10**, 17295-17300 (2018).
12. Shen, H. et al. Visible quantum dot light-emitting diodes with simultaneous high brightness and efficiency. *Nat. Photonics* **13**, 192-197 (2019).
13. Li, X. et al. Quantum-dot light-emitting diodes for outdoor displays with high stability at high brightness. *Adv. Opt. Mater.* **8** (2020).
14. Ba, G. et al. Quantum dot light-emitting diodes with high efficiency at high brightness via shell engineering. *Opt. Express* **29**, 12169-12178 (2021).
15. Deng, Y. et al. Solution-processed green and blue quantum-dot light-emitting diodes with eliminated charge leakage. *Nat. Photonics* **16**, 505-511 (2022).
16. Fan, X. et al. An efficient green-emitting quantum dot with near-unity quantum yield and suppressed auger recombination for high-performance light-emitting diodes. *Chem. Eng. J.* **461**, 142027 (2023).
17. Liu, G., Zhou, X. & Chen, S. Very bright and efficient microcavity top-emitting quantum dot light-emitting diodes with Ag electrodes. *ACS Appl. Mater. Interfaces* **8**, 16768-16775 (2016).
18. Lee, T. et al. Bright and stable quantum dot light-emitting diodes. *Adv. Mater.* **34**, 2106276 (2022).
19. Tang, Z. et al. High performance, top-emitting, quantum dot light-emitting diodes with all solution-processed functional layers. *J. Mater. Chem. C* **5**, 9138-9145 (2017).
20. Lee, T. et al. Highly efficient and bright inverted top-emitting InP quantum dot light-emitting diodes introducing a hole-suppressing interlayer. *Small* **15**, e1905162 (2019).
21. Cai, L. et al. High-efficiency top-emitting green perovskite light emitting diode with quasi lambertian emission. *Adv. Opt. Mater.* **10**, 2101137 (2021).
